# A start codon mutation of the *TSPAN12* gene in Chinese families causes clinical heterogeneous familial exudative vitreoretinopathy

**DOI:** 10.1002/mgg3.948

**Published:** 2019-08-26

**Authors:** Wei Li, Ziwei Wang, Yan Sun, Zhuoshi Wang, Jinyue Bai, Bo Xing, Xiao Sun, Lusheng Wang, Jiankang Li, Wei He

**Affiliations:** ^1^ BGI Education Center University of Chinese Academy of Sciences Shenzhen China; ^2^ He's university Shenyang China; ^3^ Shenyang He Eye Specialist Hospital Shenyang China; ^4^ School of Basic Medicine Qingdao University Qingdao China; ^5^ Department of Computer Science City University of Hong Kong Kowloon Hong Kong

**Keywords:** clinical heterogeneous manifestations, Familial exudative vitreoretinopathy, fundus fluorescein angiography, start codon mutation

## Abstract

**Background:**

Familial exudative vitreoretinopathy (FEVR) is a severe clinically and genetically heterogeneous retinal disorder characterized with failure of vascular development of the peripheral retina. The symptoms of FEVR vary widely among patients in the same family, and even between the two eyes of a given patient. The purpose of this study was to investigate the molecular mechanisms by which the start codon mutation of the *TSPAN12* causes difference in clinical manifestations between individuals in the same family.

**Methods:**

Next‐generation sequencing (NGS)‐based target capture sequencing was performed in proband with a diagnosis of FEVR and their normal visual acuity family members. Cosegregation analysis of the candidate causative variant was performed in additional family members by using Sanger sequencing. Complete fundus examination, fundus fluorescein angiography (FFA), and family history collection were performed in all family members. Potential candidate causative variants were verified with reference to guidelines and standards from the American College of Medical Genetics and Genomics.

**Results:**

We identified a novel heterozygous missense mutation (c.1A>G, p.M1V) localized in the start codon of the *TSPAN12* and was detected as a potentially disease‐causing variant for the proband. Retrospective analysis of clinical data, fundus examination, and FFA showed that the mutant carrier presented peripheral retinal vascular anomalies in early stages, and visual acuity did not show significant effects. However, the proband who carried this mutation and his cousin showed typical high‐stage FEVR fundus changes coupled with a sharp decline in vision.

**Conclusions:**

We report a novel start codon mutation (c.1A>G, p.M1V) in the *TSPAN12* that causes clinically heterogeneous manifestations. Our results expand the mutation spectrums of *TSPAN12*, and will be valuable for disease diagnosis, prognosis, genetic counseling, and enriching our understanding of the role of the tetraspanin‐12 protein in the pathogenesis of FEVR.

## 1 BACKGROUND

Familial exudative vitreoretinopathy (FEVR) is a clinically and genetically heterogeneous disorder that is one of the main causes of tractional retinal detachment and blindness in adolescents. It was first discovered and proposed by Criswick and Schepens in 1969, and since then domestic and foreign researches on FEVR have gradually deepened (Criswick & Schepens, 1969). The clinical manifestations of the disease are binocular disease, which is characterized by abnormal vascular dysplasia around the retina with neovascularization, retinal hemorrhage, retinal folds, vitreous leakage, vitreous, and retinal adhesion; stages of severity can lead to blindness (Canny & Oliver, 1976; van Nouhuys, 1991; Ober, 1980). FEVR has a strong clinical heterogeneity with disease phenotype ranging from asymptomatic to total blindness. There may be clinical symptom differences between different patients from the same family. Even the phenotype between the two eyes of a given patient may be different, which brings great difficulties in the diagnosis and intervention of the disease. Depending on clinical manifestations and fundus fluorescein angiography (FFA) diagnosis, combined with genetic testing, can help diagnose the genetic stages of FEVR families (Pendergast & Trese, 1998; Robitaille et al., 2002; Gregoryevans et al., 2004).

Genetic factors play a major role in the pathogenesis of FEVR, which often has strong familial characteristics. Previous studies have found that FEVR has a variety of inheritance patterns, with strong clinical and genetic heterogeneity. Its genetic patterns include autosomal dominant, autosomal recessive, or X‐linked recessive inheritance patterns. Numerous genetic methods show the complexity of the pathogenic mechanism; so, it is necessary to find additional pathogenic genes or disease‐causing mutations, and to study the pathogenesis of FEVR in depth, which could provide a powerful research basis for intervention and treatment of the disease. Among them, autosomal dominant inheritance is the most common form in FEVR (Berger et al., 1992). To date, *FZD4*(MIM, 604579, AD), *LRP5*(MIM 603506, AD, or AR), *TSPAN12*(MIM 613138, AD)*, KIF11*(MIM 148760, AD), *NDP*(MIM 300658, XLR), *ZNF408*(MIM 616454, AD) have been implicated in FEVR (Deng et al., 2013; Prasov et al., 2012; Rao et al., 2017). Among the genes associated with FEVR, the genetic pattern of the first three genes is autosomal dominant, and the high probability of detection of disease‐causing mutations in Korean population is 35.3%, of which TSPAN12 mutation accounts for 5.6% (Seo et al., 2016, 2015).

The protein encoded by the *TSPAN12* is located in the Wnt/Norrin‐β‐catenin signaling pathway and has a low mutation frequency in the Chinese population. The Wnt/Norrin‐β‐catenin signaling pathway plays a very important role in tissue development and cell differentiation, and is involved in cell proliferation, apoptosis, differentiation, vascular development, embryonic development, and is closely related to angiogenesis (Xu et al., 2004). There are three modes of Wnt signaling pathway, in which the classical Wnt signaling pathway (Wnt‐β‐catenin signaling pathway) is associated with FEVR. The Wnt‐β‐catenin signaling pathway is highly conserved in evolution and plays a vital role in many life activities. Norrin protein is one of the many Wnt proteins. The Norrin‐β‐catenin signaling pathway belongs to the Wnt‐β‐catenin signaling pathway and plays an important role in the development of retinal blood vessels. The inactivation of this signaling pathway leads to retinal blood vessels of dysplasia (Gilmour, 2015; Hulin‐Curtis, Williams, Wadey, Sala‐Newby, & B., & George, Sarah J., 2017; Musada, Syed, Jalali, Chakrabarti, & Kaur, 2016). *TSPAN12* is a coactivator of Norrin signaling in the retina, controlling the development and maintenance of the blood–brain barrier (BBB) and the blood–retinal barrier (BRB). It is also biologically active in BBB maintenance, but its efficacy is lower than that of Norrin. In the case of Norrin signaling, *TSPAN12* is a member of the tetraspanin family of integral membrane proteins and is required for normal retinal angiogenesis (Wang et al., 2018).

In this study, we performed direct sequencing analysis on DNA samples of FEVR family members based on targeted gene region capture protocols to find pathogenic mutations. First, the genomic DNA from the peripheral blood of patients and relatives in the family was prepared, and then primers were designed to amplify the exon and exon–intron junction regions of 790 genes associated with eye diseases including FEVR disease‐causing genes. Next‐generation sequencing (NGS)‐panel sequencing and data analysis captured different genetic variants and identified point mutations using Sanger sequencing. Here, we identified a novel initiation codon mutation c.1A>G (p.M1V) in exon 2 of *TSPAN12* in a Chinese family that caused different stages of clinical manifestations. This mutation was not present in the 1000 Genome Database (1000G), Exome Aggregation Consortium (ExAC), Single Nucleotide Polymorphism (dbSNP), and in‐house data from previous studies (Altshuler, Durbin, Abecasis, Bentley, & Cartwright, 2010; Consortium et al., 2016; Ruderfer et al., 2016; Sherry et al., 2001). Bioinformatics analysis indicated that this mutation site is critical for protein function, and multiple alignment sequence analysis indicated that the mutation is highly conserved among multiple species. This study expands the genetic database of FEVR disease in China and provides a basis for molecular diagnosis of the disease.

## 2 METHODS

### 2.1 Subjects

The study followed the Helsinki Declaration and was approved by the Ethics Committee of the Shenyang He Eye Specialist Hospital, He's university. Informed consent was signed by all patients and their families and 100 control subjects, with the underaged subjects' guardians signed the informed consent.

The medical records of all participants were collected from 2012 to 2018, and the probands were diagnosed with FEVR at Shenyang He Eye Specialist Hospital. Fluorescein Fundus Angiography was performed in participant by intravenous injection of fluorescent dyes.

### 2.2 Diagnostic criteria

The diagnostic criteria for FEVR are as follows: (1) fundus examination shows avascular around the peripheral retina; (2) FFA shows peripheral retina without blood vessels, and different degrees of subretinal exudation or retinal detachment can be found; (3) children with no preterm birth, low birth weight, and history of oxygen inhalation have a high degree of suspicion of FEVR when their symptoms are similar to retinopathy of prematurity(ROP); (4) positive family history can help diagnose; (5) exclusion of Norrie disease and ROP. The proband and his available family members were assessed by a single retinal specialist (Kashani, Brown, et al., 2014).

Based on fundus changes and FFA finding, the patients were classified into five stages using the Trese's Staging System for FEVR, which was proposed by Pendergast and Trese in 1998 (Kashani, Brown, et al., 2014; Pendergast & Trese, 1998).

This stage system was proposed for classification of the clinical and angiographic features of FEVR. Stage 1 presents avascular retinal periphery and is based on the angiographic diagnosis. Stage 2 shows avascular retinal periphery combined neovascularization with (2B) or without (2A) the clinical appearance of exudate or angiographic appearance of leakage in the late phase. Stage 3 shows extramacular retinal detachment combined with tractional or exudative. Stage 3A shows the absence of any clinical signs of exudation, subretinal fluid, leakage on angiography, whereas stage 3B has one or more of these features. Stage 4 shows subtotal macula‐involving retinal detachment combined with (4B) or without (4A) the presence of exudation. Stage 5 shows total retinal detachment. According to Trese's Staging System, two patients exhibited with typical symptoms were staged into severe FEVR (stage 3–5), four individuals with mutant carrier were staged into mild FEVR (stage 1–2).

### 2.3 Clinical evaluation

We recruited eight immediate members of this family, including six individuals with clinical diagnosis of typical FEVR. Among them, two individuals were classified into severe stages (stage 3–5) and four individuals were classified into mild stages (stage 1–2). All medical records and family history were reviewed, with full‐term delivery and normal birth weight. Comprehensive ophthalmic examination was performed in all individuals, including vision measurements, slit lamp examination, B‐ultrasound, detailed fundus photography, and FFA. The diagnosis of FEVR is done based on the clinical staging criteria described by previous report.

Fluorescein fundus angiography (FFA) is an ophthalmic examination technique that was developed in the 1960s. It can dynamically observe the circulation structure of the fundus, and has served in the diagnosis, treatment, and observation of fundus diseases since its use. With the further promotion of FFA in clinical practice, FFA plays an increasingly important role in the diagnosis of FEVR, determining the extent of lesions, and suggesting the development of the disease (Canny & Oliver, 1976). Based on the importance of FFA in the clinical diagnosis of FEVR, we require that each proband's biological parents be required to use the ophthalmic imaging platform (Spectralis HRA2; Heidelberg Engineering GmbH) for FFA to assess whether they have the stage of clinical manifestations and disease of FEVR.

### 2.4 Targeted gene capture and next‐generation sequencing

Customized gene capture chip‐based NGS was designed to encompass all the coding exons, flanking intronic regions, and untranslated regions (UTRs) of 792 genes involved in common inherited eye diseases (Table S1). The Target_Eye_792_V2 chip was custom designed and produced by MGI (MGI‐Shenzhen,China) (Gao et al., 2019). The genomic DNA sample of the proband and member in each family was subject to analysis using panel‐based NGS. Whole blood from recruited members of this study was stored in an EDTA blood collection tube and genomic DNA was extracted using FlexiGene DNA Kit (Qiagen, Venlo, The Netherlands) according to standard manufacturer's protocols. Polymerase chain reactions were done using custom primers targeting all open reading frames and the flanking intronic sequences for direct sequencing on genetic sequencer. The DNA fragment was amplified by PCR and hybridized to a DNA capture probe specifically designed for the target gene. The captured DNA fragment was eluted, amplified again, and NGS was performed using a sequencing system on Illumina HiSeq2500 Platform (Illumina; Inc.).

### 2.5 Sequence analysis and bioinformatics analysis

Target capture high‐throughput sequencing parameters are as follows: (1) target gene number includes 792 ophthalmic associate disease‐causing genes; (2) target region coverage reached 99.96%; (3) average sequencing depth is greater than 300×; (4) the average depth of the locus in the target region more than 30× accounted for 98.72%.

The sequenced reads were mapped to the human reference genome (hg38) with the Burrows‐Wheeler aligner version (BWA‐MEM; version 0.7.10). Variants calling was performed using Genome Analysis Tool Kit (GATK, Version 3.3). All variants were annotated by Annovar and SnpEff (Cingolani et al., 2012; Li & Durbin, 2009; Wang, Li, & Hakonarson, 2010). Then, the variants identified through the above pipeline were further filtered to eliminate benign variants with minor allele frequency (MAF) >0.1% in 1000 Genomes, dbSNP, EXAC, ESP6500 database, and in‐house database. Finally, combined with total depth, quality scores, MAF, potential deleterious effects, and mutation reports in common databases for mutation prioritization and selection, such as the Human Gene Mutation Database, Retinal Information Network, ClinVar, and Online Mendelian Inheritance. Man assessing variants calling for confidence (Amberger, Bocchini, Schiettecatte, Scott, & Hamosh, 2015; Daiger, Sullivan, Bowne, & Rossiter, 2013; Krawczak et al., 2015; Landrum et al., 2014). Any variant with MAF higher than 0.005 (for potential recessive variants) or 0.001 (for potential dominant variants) is considered a sequence polymorphism and will not be further analyzed. Those that passed the filter were then subjected to the following in silico analyses: SIFT, MutationTaster, Polyphen2 HDIV, Polyphen2 HVAR, Mutation Assessor. Potential deleteriousness was evaluated according to the standards and guidelines of the American College of Medical Genetics and Genomics (ACMGG).

### 2.6 Sanger validation

Primer3 was utilized to design all polymerase chain reaction primers for validation. Sanger sequencing was utilized to validate the identified mutations. Segregation studies were performed within the family members. PCR primers were designed with Primers3, the sequences of exon 2 of the *TSPAN12* were as following: 5'‐GGG‐GAC‐AGA‐CCA‐AAT‐GAT‐GT‐3', and 5'‐AAA‐CCA‐TGC‐TGC‐CTC‐AAA‐AC‐3'.

## 3 RESULTS

### 3.1 Clinical manifestations

**Figure 1:**
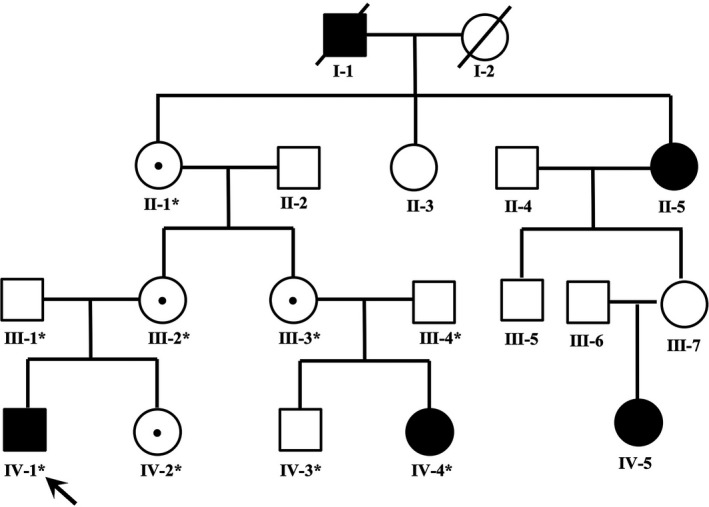
Pedigrees of the families with mutations. Squares indicate men and circles women; black and white symbols represent affected and unaffected individuals, respectively. The proband is marked with an arrow, the dotted symbols indicate carried a mutation of c.1A>G in *TSPAN12*. The asterisks indicate those members enrolled in this study

The study recruited probands and members of their families who were clinically diagnosed with FEVR from October 2012 to April 2018 at He Eye Specialist Hospital, Shenyang, China. We recruited eight members of this family, two of them were clinically diagnosed with typical FEVR performance and staged into severe FEVR (stage 3–5). The full‐field fundus and FFA examination showed a typical FEVR phenotype, of which the proband was extremely serious. Potential genotype–phenotype correlation was analyzed, and the pedigree indicated that the FEVR was inherited from an AD manner (Figure 1).

**Figure 2:**
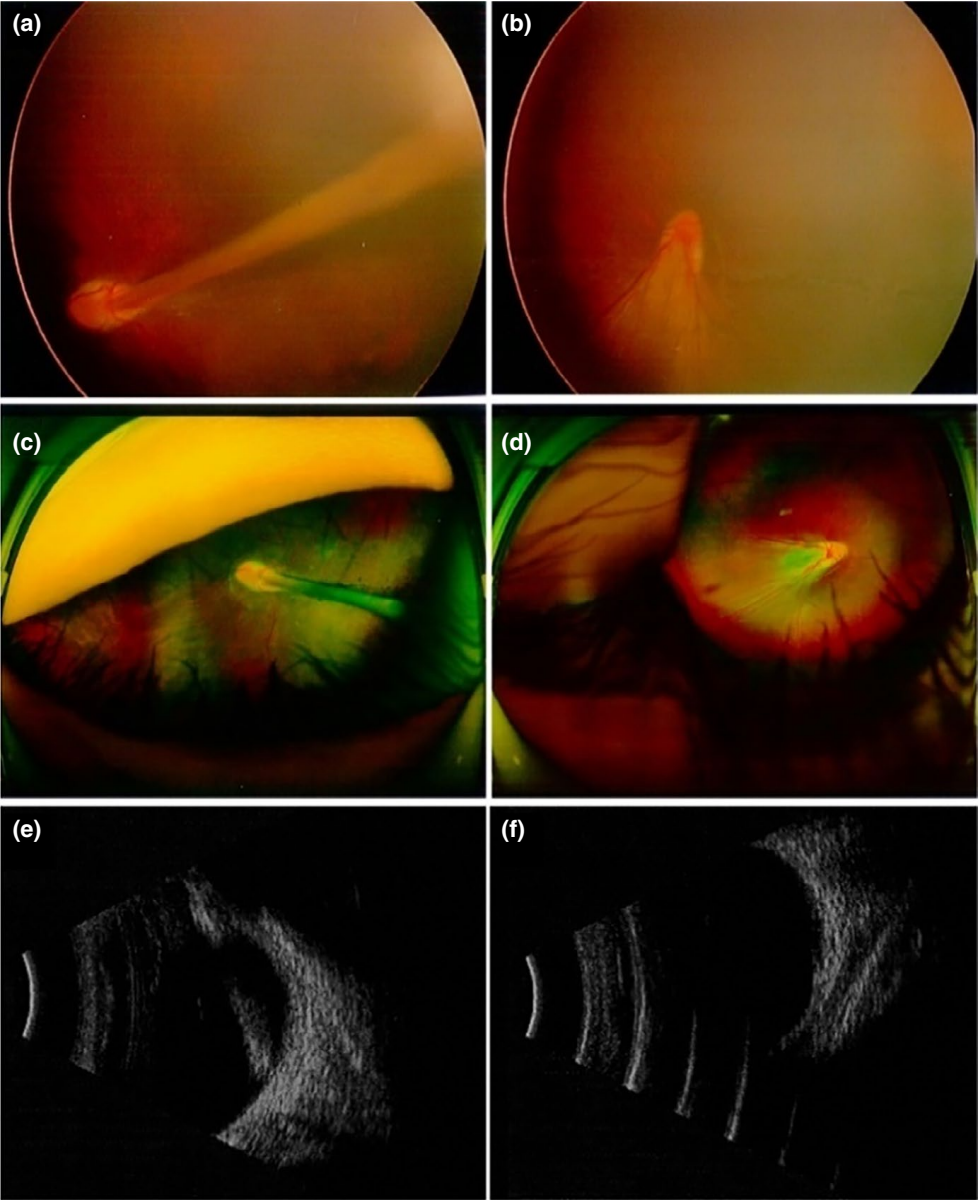
Subject IV‐1: (a, c, e) represent left eye, (b, d, f) represent right eye. B‐ultrasound, respectively, shows the scattered light spot in the vitreous cavity of the right eye, the eyeball wall of the temporal disc is not smooth, and the short light band in front of the nipple; the denser light spot in the vitreous cavity of the left eye, and the thick light echo from the nipple to the front (e, f)

**Figure 3:**
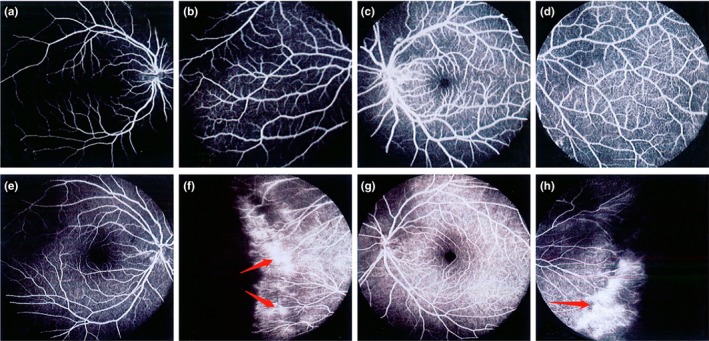
Subject III‐1: (a, b) represent right eye; (c, d) represent left eye. Fluorescein fundus angiography showed normal appearance of the posterior pole and peripheral retinal vessels of the right eye and left eye of the proband's father (III‐1), respectively; Subject III III‐2: (e, f) represent right eye; (g, h) represent left eye. (e, g) Respectively showed that the mother (III‐2) of the proband had normal fundus of the posterior pole of the right eye and the left eye. (f, h) Respectively showed that the mother of the proband had abnormal manifestations of peripheral retinal vessels in the right eye and left eye. Fluorescent fundus angiography showed no perfusion area in the peripheral retina, and fluorescence leakage appeared in the peripheral blood vessels of the retina

The heterozygous change c.1A>G (p.M1V) was found in a 7‐year‐old boy (IV‐1) who was diagnosed with FEVR at birth, manifested with typical peripheral retinal degeneration and macular dragging, tractional retinal detachment, retinal fold in both eyes. In addition, the proband had features of combined mild form of posterior persistent hyperplastic primary vitreous (PHPV) (Goldberg, 1997; Pollard, 1997) with FEVR formation, and a fibrovascular membrane extending from the optic disc to equator in left eye or parallel to the peripheral retina of the right eye (Figure 2). His mutation‐carrying mother and sister (IV‐2 and III‐2) manifested bilateral normal visual acuity with characteristics of peripheral avascular retinal combined vessel exudate and neovascularization. His father (III‐1) manifested normal visual acuity and fundus and did not carry this mutation (Figure 3). The cousin (IV‐4) is a 2‐year‐old girl who has been diagnosed with a clinical history of FEVR by a fundus specialist and manifested with poor visual acuity in the right eye. The subject was too young to get a detailed fundus examination report, but from a comprehensive clinical assessment, the overall situation is better than proband, the fundus examination report not shown. Her mother (III‐3) and grandmother (II‐1) showed typical fundus changes through FFA examination, manifested with peripheral retinal avascular and staged into mild FEVR (stage 1). Both of them have mutation‐carrying characteristics with normal visual acuity. All of the above mutation carriers underwent complete fundus examination and fluorescence fundus angiography and were diagnosed by the single clinical fundus specialist. The inspection report shows that for different individuals within the family carrying the same mutation, they can manifest with different fundus change and can be staged into mild or severe FEVR based on the Trese's Staging System.

To better describe the phenotype of FEVR in this family, we classify recruiting members into different stages based on fundus changes and FFA reports (Table S2). We found that the proband (IV‐1) and his cousin (IV‐4) developed total or subtotal retinal detachment during early childhood. According to the criteria of Trese's Staging System, the proband and his cousin were staged into stage 5 and stage 4, respectively. His mutation‐carrying (II‐1, III‐2, III‐3, IV‐2) family member showed the clinical manifestations with stage 1 or 2, but the visual acuity was not significantly affected. In addition, the fathers (III‐1, III‐4) of the two children and son (IV‐3) underwent a complete examination to determine that they have normal vision and did not carry the mutation.

### 3.2 Genotype–phenotype correlations

To investigate the genotype–phenotype relationship between causative genes and FEVR symptoms, we recruited affected asymptomatic individuals with start codon mutations in this family for genotype–phenotype analysis. We notice that the carriers of the *TSPAN12* mutation exhibit a broader phenotype profile, differing from stage 1 to stage 5. Our results showed that multiple individuals with the same mutation exhibit diverse phenotypic differences, and that carriers within the same family range from asymptomatic to almost light perception, reflecting a high degree of clinical heterogeneity.

### 3.3 Mutation identification

According to the following criteria, we consider the variation to be “pathogenic”: (1) excluding variants identified in 1000G_EAS, EXAC, SNP database (dbSNP), and 100 normal visual individuals; (2) exclusion of benign variation from ACMG and six bioinformatics methods (Table 1); (3) exclusion of families that cannot confirm to be cosegregated with FEVR.

**Table 1:** Summary of deleteriousness prediction methods analyzed mutation c.1A>G of *TSPAN12* in our study

Name	Category	Score	Deleterious threshold	Information used
SIFT	Function prediction	0	<0.05	Protein sequence conservation among homologs
PhyloP	Conservation score	3.243	>1.6	DNA sequence conservation
FATHMM	Function prediction	4.56	≥0.45	Sequence homology
PolyPhen−2 HDIV	Function prediction	0.993	>0.5	Eight protein sequence features, three protein structure features
PolyPhen−2 HVAR	Function prediction	0.971	>0.5	Eight protein sequence features, three protein structure features
MutationTaster	Function prediction	1	>0.5	DNA sequence conservation, splice site prediction, mRNA stability prediction and protein feature annotations

We identified that a novel unreported start codon mutation (c.1A>G, p.M1V) is located in exon 2 of the *TSPAN12*. Studies have shown that when the codon AUG is replaced by a GUG, the translation efficiency of the mRNA is greatly reduced. This mutation causes the methionine encoded by the initiation codon to become valine, which may greatly influence the efficiency of the encoded protein, eventually leading to a reduction in protein product causing disease. Compared to GUG, the initiation codon AUG can regulate the expression level of the protein to increase the efficiency of translation initiation (Hecht et al., 2017). This mutation is not available in the 1000 genome (G1000), EXAC, and internal databases. Multiple bioinformatics analysis software has shown that this mutation has an effect on protein function changes. Depending on the standards and guidelines of American College of Medical Genetics and Genomics (ACMGG), we assessed this mutation as a disease‐causative mutation.

### 3.4 Variant evaluation

**Figure 4:**
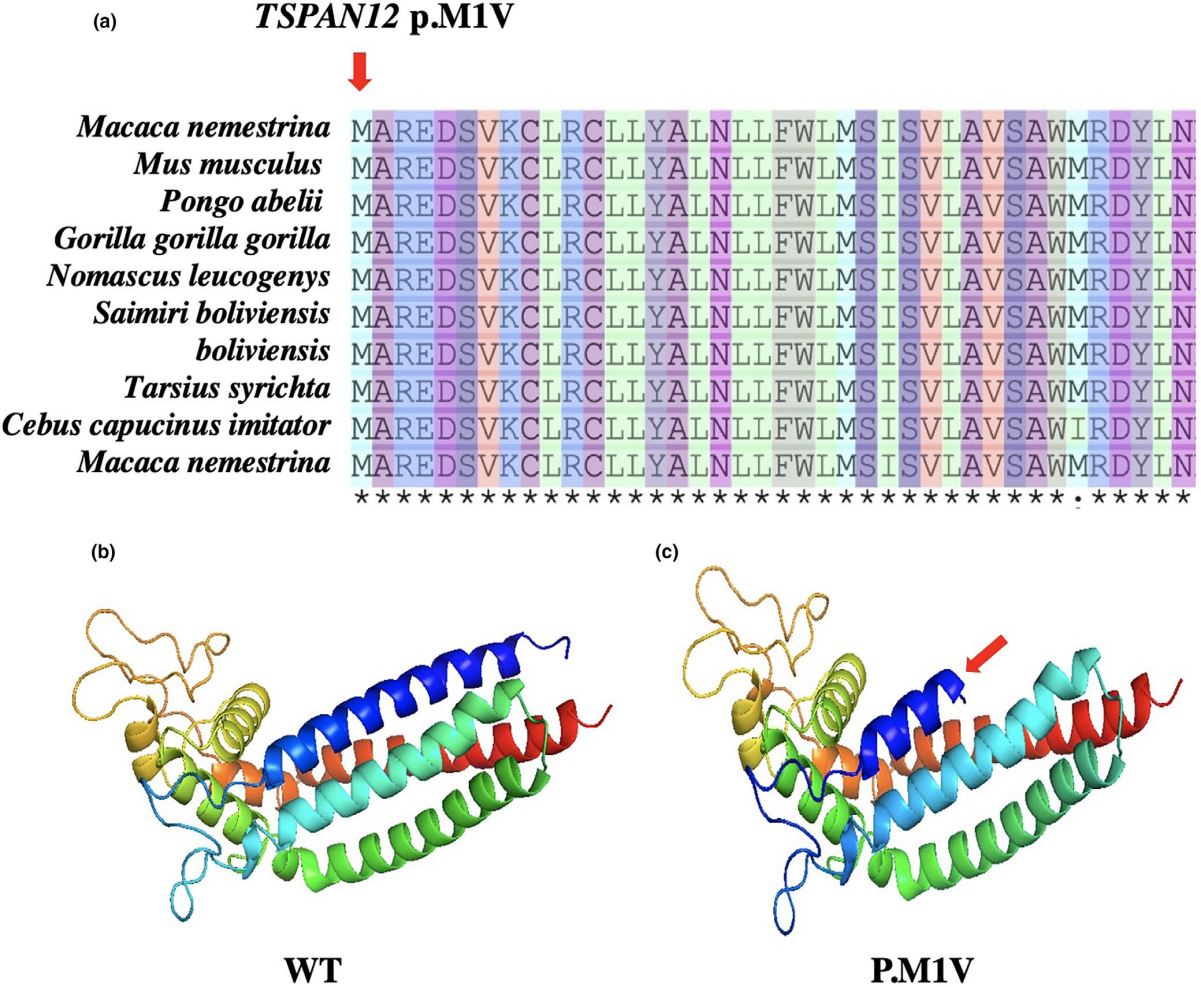
Multiple sequence alignment of start codon mutations from different species to explore the conservation of these mutations. As shown in (a), the red arrow represents mutation sites. To evaluate the influences of mutations in tetraspanin‐12, the simulation program SWISS‐MODEL was used to predict the 3‐D structure of both the mutant and wild‐type proteins. As shown in (b and c), we observed a difference between the structure of the mutated and wild‐type in tetraspanin‐12, red arrow indicates a truncated protein structure change, which resulted in a functional abnormality change

The amino acid sequence was available from the National Center for Biotechnology Information and multiple protein alignments sequence was generated using ClustalW2. Sequence flags were generated by WebLogo3. Multiple orthologous sequence alignments (MSA) indicate that the initiation codon methionine of *TSPAN12* and its subsequent sequences are highly conserved amino acids across different species. To evaluate the influences of mutations in tetraspanin‐12, the simulation program SWISS‐MODEL was used to predict the 3‐D structure of both the mutant and wild‐type proteins. We observed a significant difference between the structure of the mutant and the wild‐type in tetraspanin‐12, which produced a truncated protein structural change that led to a functional abnormality change (Figure 4). Identified mutation of all participants was confirmed by Sanger sequencing (Figure S1).

## 4 CONCLUSION

FEVR is a congenital hereditary eye disease with low incidence, which is defined as a group of inherited disease with abnormal retinal angiogenesis leading to incomplete vascularization of the peripheral retina. The affected individual may not present obvious symptoms and sign in the early stage, but severe vitreous hemorrhage and traction retinal detachment may occur in the later stages, resulting in decreased vision or even blindness. Management of FEVR depends on the clinical stage of the disease. Due to the consequences of neovascularization on visual outcomes, an early diagnosis can be vision preserving. One study showed that 58% of asymptomatic family members had clinical and angiographic evidence of stages 1 and 2 FEVR (Kashani, Learned, et al., 2014). The clinical manifestation of patients in this study varied from asymptomatic to retinal detachment. As for the mutant carriers who did not show clinical symptoms, fluorescein fundus angiography was used to observe fundus vascular change combined with genetic testing, which can significantly improve the diagnosis rate of disease in early stage compared to making diagnostic decisions based on the visual performance and family genetics information.

The novel start codon mutation c.2T>C (p.M1T) was previously reported in a 17‐year‐old male patient. He was observed to have FEVR‐associated rhegmatogenous retinal detachment in his right eye, and falciform retinal fold was observed in his left eye. His asymptomatic father manifested with typical FEVR characteristics with bilateral peripheral avascularization, but no DNA sample was available for mutational analysis (Tang, Sun, Hu, Yuan, & Ding, 2017). Our clinical findings are similar to previous reports. The proband manifested with typical peripheral retinal degeneration, macular dragging, tractional retinal detachment, and retinal fold from early childhood. Moreover, the proband had features of fibrovascular membrane extending from the optic disc to equator in left eye or parallel to the peripheral retina of the right eye. His asymptomatic mother and sister (IV‐2 and III‐2) manifested bilateral normal visual acuity with characteristics of peripheral avascular retinal combined exudate and neovascularization. However, for the first time, we performed detailed mutation analysis and clinical examinations on family members, the results showed that asymptomatic mother and sister carried the same start codon mutation as the proband. We have for the first time described the clinical heterogeneity of the *TSPAN12 *start codon mutation and its association with FEVR disease and PHPV, which is important for further understanding of the genotype–phenotype.

Here, we report a novel start codon mutation c.1A>G in the *TSPAN12* that causes affected individuals to show different stages of clinical manifestations. In order to elucidate the genotype–phenotype correlation in FEVR, clinical examination of fundus photography and FFA was performed in all participants. We found that the proband's vision did not perform well at birth, and there was a progressive change in the next few years. The visual acuity test result was 0.5/0.3 (8 month) at the first visit, and the visual acuity gradually decreased in the following years (0.2/0.1, 4‐year‐old). When he was 7‐year‐old, the visual acuity test was 0.16/0.03. His asymptomatic mother presented with periphery retinal degeneration, which had not undergone progressive changes with good visual acuity and stable fundus overs the years. However, the proband was diagnosed with FEVR disease from 8 months of age, and the clinical examination results showed more severe fundus manifestations and progressive decline in vision. For this study, affected patients presented a broad spectrum of phenotypes, from asymptomatic to retinal detachment or retinal folds so that vision is severely affected. For the first time, we systematically described the genotype‐phenotype correlation of the FEVR family, which broadens the current mutation profile of the gene and provides insight into the complexity of the genotype‐phenotype correlation of FEVR.

Our study suggests that the patients with FEVR have a high degree of clinical heterogeneity and should not be analyzed unilaterally based on genotype or phenotype in clinical diagnosis and genetic counseling. Clinicians should consider the association between genotype–phenotype in conjunction with a comprehensive fundus examination when providing genetic counseling services to families with FEVR patients. So that we could provide a more accurate diagnosis. Mutation screening of genes known to cause FEVR will provide valuable information for diagnosis and genetic counseling, particularly for patients without a family history or atypical manifestations of the condition.

In conclusion, FEVR is a rare clinical hereditary vitreoretinopathy, which is one of the main causes of retinal detachment in young adults. Early screening and early intervention in asymptomatic patients can prevent progression. The immediate family members of the patient should be examined to facilitate disease screening and genetic counseling. Indirect ophthalmoscope fundus examination combined with FFA is the main method for diagnosing FEVR. It can be found that only the peripheral retinal vascular abnormalities are involved in the early stage, which are extremely important for early diagnosis. We systematically describe the signaling pathways and potential molecular causes of the *TSPAN12* in front of the article. We believe that the molecular mechanism of this family variability is due to abnormalities caused by vascular regression and neovascularization leading to disease pathogenesis. More in‐depth molecular mechanisms need to be further explored in the next study.

## CONFLICT OF INTEREST

The authors declare that the research was conducted in the absence of any commercial or financial relationships that could be construed as a potential conflict of interest.

## AUTHOR CONTRIBUTIONS

J.L., W.H., and W.L. conceived and designed this study. Y.S., Z.W., and W.H. recruited patients, performed clinical examinations, and interpretation. W.L., Z.W., J.B., B.X., and X.S. collected the clinical samples and clinical data. J.L., Z.W., F.C., and W.L. analyzed the sequencing data. W.L, Z.W. wrote and revised the manuscript.

## Data Availability

The datasets used and/or analyzed during the current study are available from the corresponding author on reasonable request.
