# Supplementary material for: A start codon mutation of the TSPAN12 gene in Chinese families causes clinical heterogeneous familial exudative vitreoretinopathy
Source: Mol Genet Genomic Med. 2019 Aug 26;7(10):e00948. doi: 10.1002/mgg3.948 (PMC6785457; doi:10.1002/mgg3.948)
Supplement: Supplementary file 1 [file MGG3-7-e00948-s001.docx]

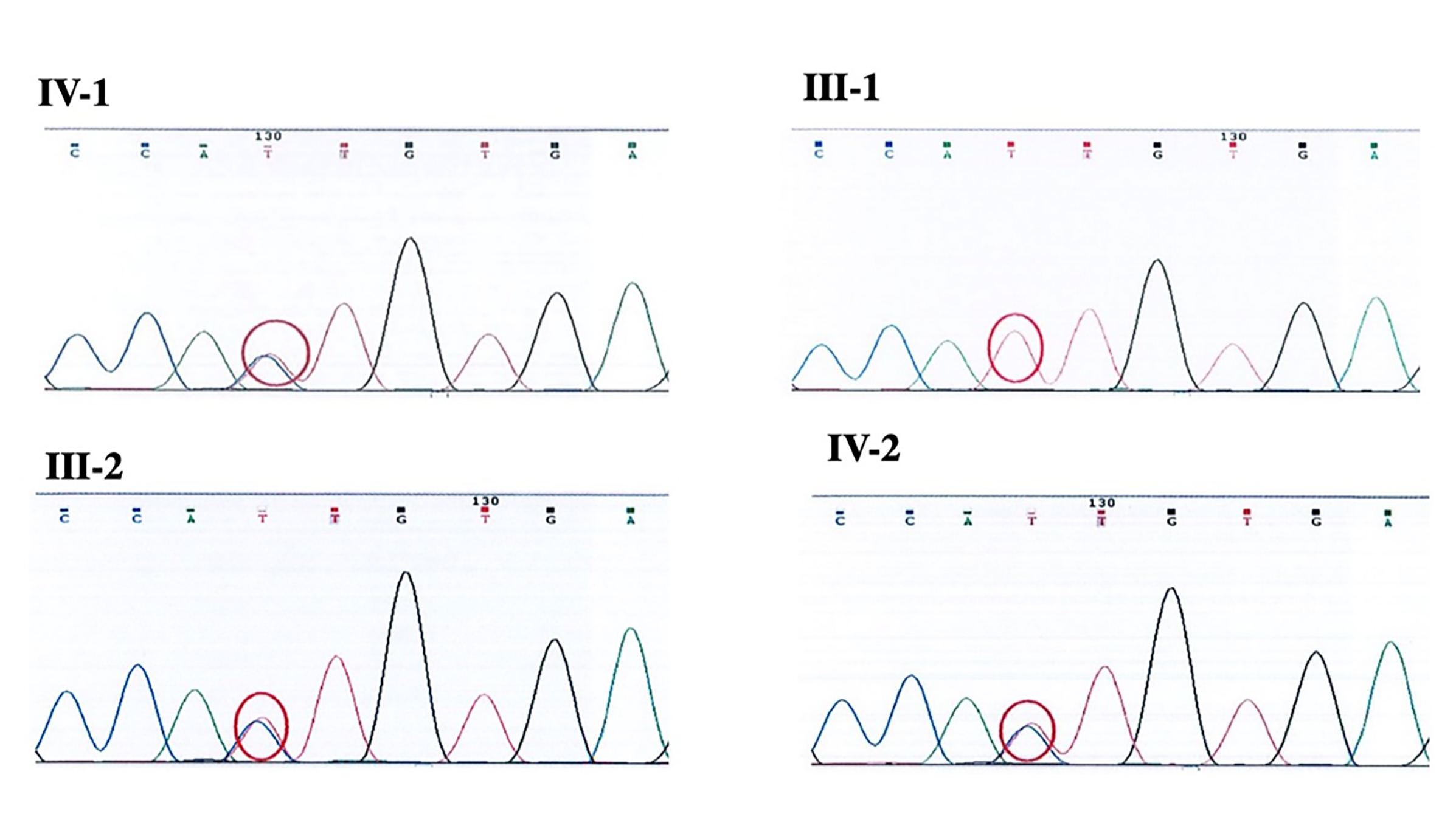


Figure S1. Sanger sequencing was used to validate the identified mutations. The sanger sequencing results showed that the father (Ⅲ-1) did not detect the mutation, and the proband (Ⅳ-1) and Asymptomatic carrier (Ⅲ-2, Ⅳ-2) detected the mutation.
