# Supplementary material for: A start codon mutation of the TSPAN12 gene in Chinese families causes clinical heterogeneous familial exudative vitreoretinopathy
Source: Mol Genet Genomic Med. 2019 Aug 26;7(10):e00948. doi: 10.1002/mgg3.948 (PMC6785457; doi:10.1002/mgg3.948)
Supplement: Supplementary file 2 [file MGG3-7-e00948-s002.docx]

**Table S1. Gene list of capture panel.**

| **All genes involved in common inherited eye diseases in the panel** |
| --- |
| SRY,ABCA4,ABCB6,ABCC2,ABCC6,ABHD12,ACBD5,ACO2,ACTA1,ACTB,ACTG1,ADAM9,ADAMTS10,ADAMTS17,ADAMTS18,ADAMTSL4,ADAR,ADGRA3,ADGRV1,ADIPOR1,AGBL5,AGK,AHI1,AIPL1,ALMS1,ANAPC1,ANO5,AP3B1,APTX,ARL13B,ARL2BP,ARL3,ARL6,ARMS2,ASRGL1,ATF6,ATP13A2,ATP2C1,ATXN10,ATXN7,B3GALNT2,B3GLCT,B4GAT1,B9D1,BAP1,BBIP1,BBS1,BBS10,BBS12,BBS2,BBS4,BBS5,BBS7,BBS9,BCOR,BEST1,BFSP1,BFSP2,BLOC1S3,BLOC1S6,BMP4,BRAF,C10orf11,C10orf2,C12orf65,C1QTNF5,C2,C21orf2,C2orf71,C  3,C5orf42,C8orf37,C9,CA4,CABP4,CACNA1F,CACNA2D4,CAPN3,CAPN5,CAV3,CBS,CC2D2A,CDH23,CDH3,CDHR1,CEP164,CEP250,CEP290,CEP41,CERKL,CFB,CFH,CFI,CFL2,CHD7,CHM,CHMP4B,CHN1,CHST6,CIB2,CISD2,CLN3,CLN5,CLN6,CLN8,CLRN1,CLUAP1,CNBP,CNGA1,CNGA3,CNGB1,CNGB3,CNNM4,COL11A1,COL11A2,COL18A1,COL2A1,COL4A1,COL6A1,COL6A2,COL6A3,COL9A1,COL9A2,CRB1,CRX,CRYAA,CRYAB,CRYBA1,CRYBA2,CRYBA4,CRYBB1,CRYBB2,CRYBB3,CRYGB,CRYGC,CRYGD,CRYGS,CSPP1,CST3,CTC1,CTDP1,CTNNA1,CTSD,CTSF,CX3CR1,CYP1B1,CYP4V2,DCN,DHDDS,DHX38,DMD,DMPK,DNA2,DNAJC5,DRAM2,DRD5,DTHD1,DTNBP1,DUX4,DYSF,EDN3,EDNRB,EFEMP1,ELOVL4,EMC1,EMD,EPHA2,ERCC1,ERCC2,ERCC6,ERCC8,EXOSC2,EYS,FAM126A,FAM161A,FBLN5,FBN1,FGF10,FGFR1,FGFR2,FGFR3,FHL1,FKRP,FKTN,FLVCR1,FOXC1,FOXE3,FOXL2,FRAS1,FREM1,FREM2,FRMD7,FSCN2,FTL,FYCO1,FZD4,GABRB1,GALC,GCNT2,DF3,GDF6,GFER,GJA1,GJA3,GJA8,GJB2,GMPPB,GNAT1,GNAT2,GNB3,GNPTG,GP1BA,GPR143,GPR179,GRIP1,GRK1,GRM6,GRN,GUCA1A,GUCA1B,GUCY2D,HARS,HCCS,HESX1,HFE,HGSNAT,HK1,HMCN1,HMGB3,HMX1,HPS1,HPS3,HPS4,HPS5,HPS6,HSF4,HTRA1,IDH3B,IDUA,IFT140,IFT172,IFT27,IGBP1,IKBKG,IMPDH1,IMPG1,IMPG2,INPP5E,INVS,IQCB1,ISPD,ITGA2B,ITGA7,ITGB3,ITM2B,JAG1,JAM3,KCNJ10,KCNJ13,KCNV2,KCTD7,KERA,KIAA1549,KIF11,KIF21A,KIF7,KIT,KIZ,KLHL7,KMT2D,KRAS,KRT12,KRT3,LAMA1,LAMA2,LAMB2,LCA5,MTND1,LIM2,LMNA,LOXL1,LRAT,LRIT3,LRP5,LTBP2,LYST,LZTFL1,MAB21L2,MAF,MAK,MAP2K1,MAPKAPK3,MAPT,MC1R,MCOLN1,MERTK,MFN2,MFRP,MFSD8,MIP,MIR204,MITF,MKKS,MKS1,MT-ATP6,MTM1,MT-TH,MT-TL1,MTTP,MT-TP,MTTS2,MVK,MYH7,MYO7A,MYOC,MYOT,NBAS,NDP,NEB,NEK2,NEUROD1,NHS,NMNAT1,NOD2,NPHP1,NPHP3,NPHP4,NR2E3,NR2F1,NRAS,NRL,NTF4,NYX,OAT,OCA2,OCRL,OFD1,OPA1,OPA3,OPN1LW,OPN1MW,OPN1SW,OPTN,OR2W3,OTX2,PABPN1,PANK2,PAX2,PAX3,PAX6,PCDH15,PCYT1A,PDE6A,PDE6B,PDE6C,PDE6G,PDE6H,PDZD7,PEX1,PEX2,PEX7,PGK1,PHOX2A,PHYH,PIGL,PIKFYVE,PITPNM3,PITX2,PITX3,PLA2G5,PLEC,PLG,PLK4,PNPLA6,POC1B,POLG,POLG2,POMGNT1,POMGNT2,POMK,POMT1,POMT2,PPT1,PRCD,PRDM13,PRDM5,PROM1,PRPF3,PRPF31,PRPF4,PRPF6,PRPF8,PRPH2,PRPS1,PRSS56,PTPN11,RAB18,RAB28,RAB3GAP1,RAB3GAP2,RAF1,RARB,RAX,RAX2,RB1,RBP3,RBP4,RCBTB1,RD3,RDH11,RDH12,RDH5,RGR,RGS9,RGS9BP,RHO,RIMS1,RLBP1,ROM1,RP1,RP1L1,RP2,RP9,RPE65,RPGR,RPGRIP1,RPGRIP1L,RRM2B,RS1,RTN4IP1,RYR1,SAG,SALL1,SDCCAG8,SEMA4A,SEPN1,SETX,SGCA,SGCB,SGCD,SGCG,SHH,SHOX,SIL1,SIX6,SLC16A12,SLC24A1,SLC24A5,SLC25A4,SLC25A46,SLC26A4,SLC45A2,SLC4A11,SLC4A4,SLC7A14,SMOC1,SNAI2,SNRNP200,SOS1,SOX10,SOX2,SPATA7,SPP2,STRA6,SYNE1,SYNE2,TACSTD2,TBC1D20,TCAP,TCOF1,TCTN1,TCTN2,TCTN3,TDRD7,TEAD1,TFAP2A,TGFBI,TIMM8A,TIMP3  ,TINF2,TLR4,TMEM126A,TMEM138,TMEM216,TMEM231,TMEM237,TMEM5,TMEM67,TNNT1,TOPORS,TP63,TPM2,TPM3,TPP1,TREX1,TRIM32,TRIM37,TRNT1,TRPM1,TSPAN12,TTC21B,TTC8,TTLL5,TTN,TTPA,TTR,TUB,TUBA8,TUBB3,TUBGCP4,TUBGCP6,TULP1,TYR,TYRP1,UBIAD1,UNC119,USH1C,USH1G,USH2A,VAX1,VCAN,VIM,VSX1,VSX2,WDPCP,WDR19,WDR36,WFS1,ZNF408,ZNF423,ZNF469,ZNF513,ABCA3,ABHD5,ACD,ACVRL1,AFG3L2,AGPS,AGRN,AGXT,ALDH18A1,ALDH1A3,,ALDH3A2,ANO10,ANTXR1,AP4M1,AP5Z1,AP  C,ARSB,ARSE,ASAH1,ASB10,ASPM,ATL1,ATM,ATP6V0A2,ATP7A,ATP7B,AUH,B4GALNT1,BLM,C19orf12,CCM2,CDK5RAP2,CENPJ,CEP135,CEP152,CHAT,CHMP1A,CHRNG,CLCN7,COASY,COL3A1,COL4A4,COL4A5,COLEC11,COLQ,COX10,COX15,COX7B,CREBBP,CTSA,CUBN,CYLD,CYP27A1,CYP2U1,CYP7B1,DAG1,DBH,DDHD2,DDX59,DHCR7,DHODH,DNAJC19,DNM2,DOK7,EBP,EDARADD,EEF2,EFEMP2,EP300,EPG5,ERCC3,ERLIN2,ESCO2,EXOSC3,FA2H,FAH,FLNA,FMR1,FUCA1,GALE,GALK1,GALNS,GALT,GBA,GBA2,GCM2,GDF2,GFAP,GLB1,GM2A,GNAS,GNPAT,GNS,GRHPR,GUSB,HDAC8,HEXA,HGD,HOGA1,HPD,HSPD1,HSPG2,HYAL1,IDS,IKBKAP,IRF6,ITPR1,KCNC3,KCND3,KCNH2,KCNJ2,KCNQ1,KDM6A,KIAA0196,KIF1A,KIF1BP,KIF5A,LAMA3,LAMB3,LAMC2,LARGE1,LCAT,LMX1B,LRPAP1,LTBP4,MAN2B1,MANBA,MARS2,MCPH1,MID1,MLPH,MMACHC,MRE11A,MSH2,MTPAP,NAGLU,NEU1,NF2,NFIX,NHP2,NIPA1,NIPBL,NOP10,NOTCH2,NPC1,NPC2,NSD1,OSTM1,PDK3,PEX10,PEX11B,PEX12,PEX13,PEX14,PEX16,PEX26,PEX5,PEX6,PHF6,PIK3R1,PKP1,PLA2G6,PLOD1,POLR1C,PORCN,PRIMPOL,PRKCG,PRX,PTCH1,PTCH2,PTH,PYCR1,RAB27A,RAD21,RARS2,RECQL4,REEP1,RNASEH1,ROBO3,RTN2,SALL4,SCN4A,SEPSECS,SF3B4,SLC33A1,SLITRK6,SMC1A,SMPD1,SNX10,SOD2,SPG11,SPG7,SPINK5,SPTBN2,STIL,STK11,STS,SURF1,SYT14,TACO1,TAT,TBX1,TCIRG1,TERT,TFAP2B,TGFBR1,TGM6,TLR1,TLR2,TNFRSF11A,TNFSF11,TNXB,TRPV4,TSC1,TSC2,TSEN2,TSEN34,TSEN54,TTBK2,TWIST1,UCHL1,UROD,VHL,VRK1,WHRN,WRAP53,WRN,WWOX,XPA,XPC,ZFYVE26,ZFYVE27,ZNF335,ZNF644,ACTA2,ATP1A3,CAV1,CHRDL1,CNTN1,COL8A2,FBN2,IARS2,LONP1,MPZ,MSMO1,MSTN,MYH11,MYLK,NAA10,P3H2,PRKG1,PXDN,SEMA3E,SLC2A10,SMAD3,TCF4,TGFB2,TGFB3,TGFBR2,ZEB1 |
