# Supplementary material for: A start codon mutation of the TSPAN12 gene in Chinese families causes clinical heterogeneous familial exudative vitreoretinopathy
Source: Mol Genet Genomic Med. 2019 Aug 26;7(10):e00948. doi: 10.1002/mgg3.948 (PMC6785457; doi:10.1002/mgg3.948)
Supplement: Supplementary file 3 [file MGG3-7-e00948-s003.docx]

**Table S2. Clinical features of affected probands with variants identified in this study**

| **ID** | **Sex** | **Age at examination**  **(yrs)** | **Trese’s Staging System**  **OD/OS** | **Retinal Folds or**  **Macular Dragging**  **OD/OS** | **Visual Acuity**  **OD/OS** | **Base Change** | **Amino Acid Change** | **Frequency in Control** |
| --- | --- | --- | --- | --- | --- | --- | --- | --- |
| Ⅳ-1 | M | 6 | 3B/4B | +/+ | 0.16/0.03 | c.1A>G | p.M1V | 0/200 alleles |
| Ⅳ-2 | F | 5 | 2A/1B | -/- | 0.8/1.0 | c.1A>G | p.M1V | 0/200 alleles |
| Ⅳ-3 | M | 6 | No FEVR | -/- | 1.0/0.9 | *NA* | *NA* | *NA* |
| Ⅳ-4 | F | 5 | 4A/3B | +/+ | 0.05/0.06 | c.1A>G | p.M1V | 0/200 alleles |
| Ⅲ-1 | M | 33 | No FEVR | -/- | 1.0/1.2 | *NA* | *NA* | *NA* |
| Ⅲ-2 | F | 32 | 2B/2B | -/- | 0.7/0.8 | c.1A>G | p.M1V | 0/200 alleles |
| Ⅲ-3 | F | 30 | 1A/1B | -/- | 1.0/0.9 | c.1A>G | p.M1V | 0/200 alleles |
| Ⅲ-4 | M | 33 | No FEVR | -/- | 1.0/1.2 | *NA* | *NA* | *NA* |
| Ⅱ-1 | F | 53 | 1A/1A | -/- | 0.1/0.1 | c.1A>G | p.M1V | 0/200 alleles |

*** Phenotype–Genotype Analysis in Trese’s Staging System; Patients were staged in the basis of the highest stage of FEVR in either eye; *NA*：Not available;**
